# Intraspecific Variation in Life Cycle Impacts the Morphology of Alpine Newts

**DOI:** 10.1093/icb/icag164

**Published:** 2026-09-12

**Authors:** Morgane Fournier, Vivien Louppe, Isabelle Toussaint-Lardé, Clara Pierson, Narimane Chatar, Z Jack Tseng, Gabriel Ferreira, Christina Kyriakouli, Morgane Taillades, Lauriane Poloni, Julien Clavel, Anne-Claire Fabre

**Affiliations:** Institute of Ecology and Evolution, Universität Bern, Bern 3012, Switzerland; Naturhistorisches Museum der Burgergemeinde, Bern 3005, Switzerland; Institute of Ecology and Evolution, Universität Bern, Bern 3012, Switzerland; Naturhistorisches Museum der Burgergemeinde, Bern 3005, Switzerland; Institute of Ecology and Evolution, Universität Bern, Bern 3012, Switzerland; Naturhistorisches Museum der Burgergemeinde, Bern 3005, Switzerland; Mécanismes Adaptatifs et Evolution, UMR 7179, Muséum national d’Histoire naturelle CNRS, Paris 75005, France; Institute of Ecology and Evolution, Universität Bern, Bern 3012, Switzerland; Naturhistorisches Museum der Burgergemeinde, Bern 3005, Switzerland; Functional Anatomy and Vertebrate Evolution Lab, Department of Integrative Biology, University of California, Berkeley, 3040 Valley Life Sciences Building, Berkeley, CA 94720, USA; Functional Anatomy and Vertebrate Evolution Lab, Department of Integrative Biology, University of California, Berkeley, 3040 Valley Life Sciences Building, Berkeley, CA 94720, USA; Senckenberg Centre for Human Evolution and Palaeoenvironment at the University of Tübingen, Tübingen 72074, Germany; Geosciences Department, University of Tübingen, Tübingen 72076, Germany; Senckenberg Centre for Human Evolution and Palaeoenvironment at the University of Tübingen, Tübingen 72074, Germany; Geosciences Department, University of Tübingen, Tübingen 72076, Germany; Institute of Ecology and Evolution, Universität Bern, Bern 3012, Switzerland; Naturhistorisches Museum der Burgergemeinde, Bern 3005, Switzerland; Mécanismes Adaptatifs et Evolution, UMR 7179, Muséum national d’Histoire naturelle CNRS, Paris 75005, France; Department of Biology, Evolutionary Morphology of Vertebrates, Ghent University, Ghent 9000, Belgium; Biogéosciences, UMR 6282 CNRS, Université Bourgogne Europe, Dijon 21000, France; EPHE, PSL University, Paris 75014,France; Université Lyon 1 CNRS, UMR 5023 - LEHNA, Laboratoire d’Ecologie des Hydrosystèmes Naturels et Anthropisés, Villeurbanne 69622, France; Institute of Ecology and Evolution, Universität Bern, Bern 3012, Switzerland; Naturhistorisches Museum der Burgergemeinde, Bern 3005, Switzerland; Department of Life Sciences, The Natural History Museum, London SW7 5BD, UK

## Abstract

Facultative paedomorphosis refers to the ability of individuals within a species to reach sexual maturity either by undergoing metamorphosis (metamorph) or by retaining larval traits into adulthood (paedomorph). This developmental flexibility provides a natural framework to investigate how facultative ontogenetic pathways shape morphological variation. Here, we investigate how life cycle variation (metamorphs, paedomorphs, and paedomorphs undergoing metamorphosis) affects the size and morphology of the body and feeding apparatus (cranium, mandible, and hyoid) in the Alpine newt (*Ichthyosaura alpestris*), using both linear and geometric morphometric approaches. Our results reveal clear differentiation in size, allometric trajectories, and morphology of both the body and the feeding apparatus. Paedomorphs are smaller and exhibit morphologies consistent with aquatic functional demands and the consequences of incomplete metamorphosis, particularly in the feeding apparatus, which normally undergoes extensive remodeling during metamorphosis. Individuals undergoing metamorphosis are morphologically distinct from paedomorphs but overlap with metamorphs in body size, suggesting that metamorphosis involves rapid skeletal reorganization before post-metamorphic growth occurs. Moreover, patterns of morphological integration and modularity do not differ between life cycle categories, suggesting that the developmental interdependence of phenotypic traits does not promote the morphological changes observed in species that experience facultative paedomorphosis. Paedomorphic populations nevertheless show higher morphological disparity in the feeding apparatus, likely reflecting variation in the degree of ossification and remodeling resulting from incomplete metamorphosis. Overall, these results show that facultative life cycles strongly structure body and feeding morphology in Alpine newts, while leaving trait covariation largely unchanged. These findings emphasize the potential of developmental plasticity to generate substantial morphological diversity within a same species, providing a basis for understanding how paedomorphic lineages maintain morphological variation despite persistent developmental constraints.

## Introduction

Many newt and salamander species (Caudata) exhibit a multiphasic life cycle (Duellman and Trueb 1994; Bonett et al. 2022; Liedtke et al. 2022). In these species, eggs are laid in water and hatch into aquatic larvae. Following this aquatic phase, individuals develop into terrestrial juveniles and subsequently into adults, which generally return to aquatic habitats during the breeding season. The succession of these distinct life stages involves morphological transformations associated with ecological transition (Wilbur 1980; Moran 1994), with metamorphosis marking the shift from an aquatic larva to a terrestrial juvenile. Metamorphosis is mediated by thyroid hormones and associated with the loss of external gills and tail fin, accompanied by substantial remodeling of internal organs and skeletal structures (Rose 2003; Brown and Cai 2007). This remodeling particularly affects the feeding apparatus: some skeletal elements are resorbed (e.g., the palatine portion of the palatopterygoid, the coronoid, the larval ceratobranchials, and the urohyal bone), while others develop (the pars palatina of the premaxilla and maxilla, the vomerine tooth rows or the parasphenoid tooth patches, and the hyoid elements), expand (e.g., the vomer), or are reoriented (e.g., the pterygoid) (Rose 2003). These morphological changes are mainly associated with a shift in feeding mode accompanying the transition between environments, from aquatic suction feeding to terrestrial lingual prehension (Deban and Marks 2002).

However, in some species, changes in growth rates and developmental processes reflecting shifts in the timing or rate of development (i.e., heterochrony; Alberch et al. 1979; McKinney and McNamara 1991) can lead to incomplete metamorphosis and to the emergence of paedomorphosis. Paedomorphic individuals therefore reach sexual maturity while retaining larval traits (e.g., external gills) and remain fully aquatic (Larson 1980). Although developmental trajectories are fixed in many species, with individuals following either a metamorphic or a paedomorphic pathway, some possess the ability to express both developmental trajectories through facultative paedomorphosis. In these species, both adult morphs can coexist within the same population (Whiteman 1994; Denoël et al. 2005b).

Several hypotheses have been proposed to explain the adaptive benefits of this developmental flexibility. By reducing the energetic costs, risks (predation and/or competition), and habitat shifts associated with metamorphosis, it may confer ecological advantages while also promoting morphological differentiation among alternative phenotypes (polyphenism; Whiteman 1994; Denoël et al. 2005b). Moreover, this flexibility can broaden the range of environments that populations can exploit, providing access to different ecological niches and resources and thereby enhancing persistence under environmentally heterogeneous conditions (Denoël et al. 2005b; Whiteman 1994). It may also reduce intraspecific competition by allowing the paedomorphic and metamorphic morphs to coexist within the same breeding ponds and exploit distinct trophic niches (Lejeune et al. 2018). Importantly, in several species exhibiting facultative paedomorphosis, paedomorphic individuals can undergo metamorphosis at a later stage when environmental conditions deteriorate (Patterson 1978; Denoël 2003; Denoël et al. 2005b; Doyle and Whiteman 2008), highlighting a potential survival advantage through developmental flexibility. However, observations of paedomorphic populations of newts and salamanders are geographically restricted compared to those of metamorphic ones and appear to be declining (e.g., Denoël et al. 2001, 2023, 2019; Okamiya et al. 2021). This restricted distribution and apparent decline may partly reflect the specific conditions required for paedomorphosis to persist. Although genetic evidence suggests that the capacity to express this developmental pathway is restricted to certain populations (Semlitsch et al. 1990; Bonett et al. 2014; Denoël 2025), the persistence of the aquatic paedomorphic phenotype is also strongly dependent on local environmental conditions. In particular, paedomorphosis is favored in deep, stable aquatic habitats with low predation pressure, which provide suitable conditions for the persistence of aquatic adults (Denoël and Ficetola 2014; Lyons and Arbuckle 2024).

This strong dependence on local environmental conditions raises a complementary question: once paedomorphosis is expressed, does the retention of larval traits constrain the range of trajectories and adult phenotypes that can be produced? Such developmental constraints could differ from those acting in metamorphosing salamanders, potentially influencing the extent of morphological variation associated with alternative developmental pathways. This hypothesis has received support at the interspecific level, with Bonett and Blair (2017) showing that the body form of salamanders is subject to stronger constraints on morphological evolution in biphasic lineages than in paedomorphic ones. However, Vučic et al. (2019) demonstrated that metamorphosis does not canalize head shape variation during larval development, and that the metamorphosed phenotype is not more constrained than the larval one. These contrasting results suggest that developmental transitions do not necessarily impose uniform constraints across morphological traits.

Because alternative developmental trajectories (i.e., metamorphic versus paedomorphic pathways) directly influence how organisms grow through differences in heterochronic processes, they are expected to affect size, allometry (i.e., the size-dependent changes on shape) and ultimately adult morphology (McNamara 2012). Studying allometry is therefore essential for understanding how facultative paedomorphosis can generate morphological variation between paedomorphic and metamorphic individuals. Beyond allometry, shifts in developmental timing may affect not only the size and shape of individual traits, but also their developmental organization, including the degree of independence among them. Developmental trajectories can influence developmental organization by altering the timing and coordination of growth and differentiation among traits (Smith 2001; McNamara 2012). In particular, alternative developmental trajectories could modify the developmental relationships and covariation among morphological traits through differences in growth coordination and metamorphic remodeling (Rose 2003 ; Klingenberg 2014; Schwarz et al. 2020). Patterns of morphological integration and modularity can therefore be used to characterize how these alternative developmental trajectories shape the covariation and relative independence of morphological traits (Klingenberg 2014). Both morphological integration and modularity results from shared developmental, genetic pathways and/or function interactions: integration refers to the covariation among traits, whereas modularity describes the partitioning of traits into quasi-independent subsets (i.e., module) that exhibit stronger covariation within than between modules (Klingenberg 2008; Rolian and Willmore 2009). These patterns of covariation can influence the potential range of phenotypic variation that is accessible during development and evolution. Strong integration among traits may constrain their independent variation, thereby channeling phenotypic change along a limited number of directions, whereas a more modular organization may allow greater independence among morphological modules and facilitate changes in particular trait complexes without necessarily affecting the phenotype as a whole (Wagner and Altenberg 1996; Drake and Klingenberg 2010; Renaud et al. 2012; Goswami et al. 2014; Klingenberg 2014). In Caudata, previous studies have already shown the influence of life cycle strategy on trait covariation. Paedomorphic species tend to exhibit more integrated morphologies of the feeding apparatus, whereas metamorphic species are more modular, potentially enabling a broader range of shapes associated with broader feeding strategies (Fabre et al. 2020; Louppe et al. 2025). However, the degree of integration or modularity does not necessarily translate directy into the amount of morphological diversity (i.e., disparity) within a population. Disparity reflects the realized range of phenotypes, which results not only from the organization of trait covariation, but also from the environmental conditions experienced by individuals.

Combining analyses of allometry, modularity, integration, and disparity, we can therefore gain a clearer understanding of how intraspecific variation in life cycle type fosters morphological patterns and ultimately affects the evolutionary potential of facultatively paedomorphic species. In this context, we examine how facultative paedomorphosis shapes body size and morphological patterns, including overall body morphology and the structure of the feeding system, in the Alpine newt (*Ichthyosaura alpestris*).

The Alpine newt is a great model species for studying this phenomenon, due to its well-documented capacity for facultative paedomorphosis (Džukić et al. 1990; Whiteman 1994; Denoël et al. 2001, 2005b). Paedomorphic and metamorphic individuals represent alternative adult phenotypes within *I. alpestris* and coexist within populations (Denoël and Joly 2000). The species is widely distributed across Europe with observations of paedomorphic Alpine newt primarily concentrated in the Italian and Balkan Peninsulas. Additional records have been made in the French Alps, Switzerland, and Greece (Fig. 1A), but the species declined in recent decades (Denoël et al. 2001, 2023, 2019).

**Fig. 1:**
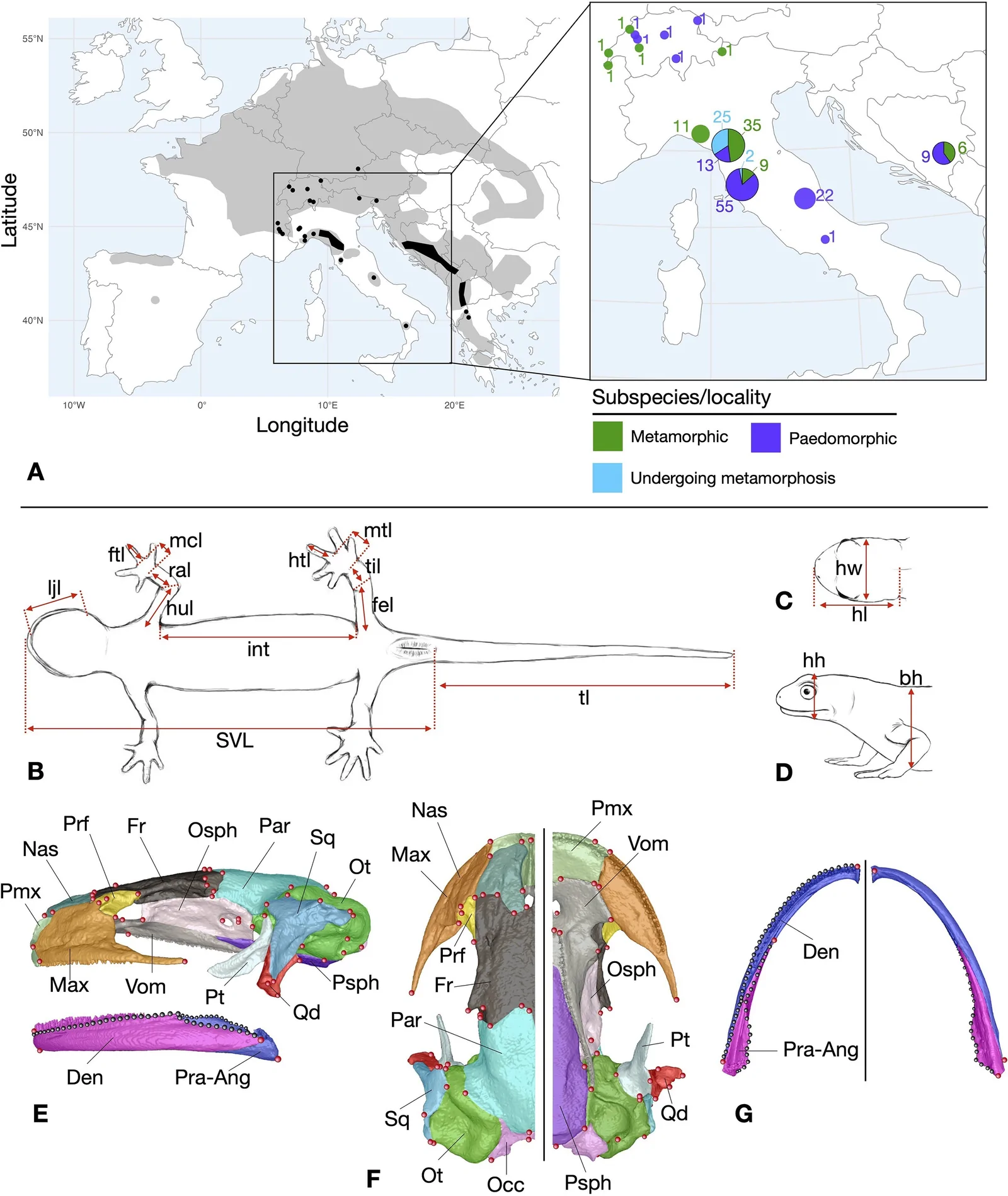
Geographic distribution of the specimens used (A), linear measurements (B–D) and landmarks (E–G) protocol. In A, the species distribution is shown in gray, whereas occurrences of facultative paedomorphosis are indicated in black. Numbers indicate the number of individuals sampled at each locality. In E–G, red dots represent type II landmarks (defined by geometric features, such as maxima of curvature or points of greatest convexity or concavity on anatomical structures) and black dots represent curve-sliding semilandmarks. Each bone is represented in different colors. In F and G, the right side of the structure is in ventral view, the left side is in dorsal view. Abbreviations are as follow: bh: body height; Den: dentary; fel: femur length; Fr: frontal; ftl: front toe length; hl: head length; hw: head width; hh: head height; htl: hind toes length; hul: humerus length; int: interlimb length; ljl: lower jaw length; Max: maxilla; mcl: metacarpus length; mtl: metatarsus length; Nas: nasal; Occ: occipital; Osph: orbitosphenoid; Ot: otic; Par: parietal; Psph: parasphenoid; Pra-Ang: prearticular-angular; Prf: prefrontal; Pmx: premaxilla; Pt: pterygoid; Qd: quadrate; ral: radius length; Sq: squamosal; SVL: snout-vent length; tl: tail length; til: tibia length; Vom: vomer.

Our first objective is to test whether paedomorphic and metamorphic individuals exhibit distinct allometric trajectories, resulting in morphological differentiation. Differences in the feeding apparatus (cranium, mandible, and hyoid) are expected to reflect both incomplete metamorphosis and adaptations to an exclusively aquatic lifestyle in paedomorphic populations. By contrast, changes in body morphology during metamorphosis tend to be less pronounced and are expected to reflect functional shifts between aquatic and terrestrial locomotor and feeding modes (Duellman and Trueb 1994). Individuals undergoing metamorphosis in response to deteriorating aquatic habitats are predicted to occupy intermediate positions in the morphological space (morphospace), reflecting progressive morphological change along a gradient of metamorphic completion associated with habitat transition. Our second objective is to assess how facultative paedmorphosis influence patterns of morphological integration, modularity, and disparity of the body and feeding apparatus. We expect paedomorphic populations to exhibit a more integrated morphology, whereas metamorphic populations are expected to show a more modular one, following patterns reported in previous interspecific studies (Fabre et al. 2020; Louppe et al. 2025). In paedomorphic lineages, the interruption of metamorphosis does not occur uniformly at the same stage in every individual causing a variation in metamorphosis completion that may increase morphological disparity despite strong trait covariation (Ivanović et al. 2014). In contrast, alternating between aquatic and terrestrial environments exposes metamorphic individuals to contrasting functional demands (Duellman and Trueb 1994; Thiesmeier and Schulte 2010; Heiss et al. 2013), which may increase body morphological disparity compared with paedomorphic individuals that remain associated with aquatic habitats.

## Material and methods

### Data sampling

Adult specimens of Alpine newts preserved in alcohol were obtained from several museum collections: Muséum d’histoire naturelle of Geneva, Naturhistorisches Museum Bern der Burgergemeinde, Naturhistorisches Museum Basel, Muséum national d’Histoire naturelle of Paris, Naturhistorisches Museum Wien, La Specola in Florence, Institut Royal des Sciences Naturelles de Belgique in Bruxelles and University of California Museum of Vertebrate Zoology in Berkeley. We sampled specimens from two subspecies across three localities in Italy, Switzerland, and Bosnia, where both metamorphic and paedomorphic life cycle occur (Fig. 1A). A total of 198 Individuals belonging to three categories were used: metamorph, paedomorph, and paedomorph undergoing metamorphosis (Fig. 1A; Table S1). Individuals undergoing metamorphosis were predominantly sampled from a single population, with only two individuals originating from one other population. Paedomorphs were identified by the presence of external gills and gill slits, together with mature cloacal morphologies (male: well-swollen cloaca without grooves; female: smaller cloaca with distinct grooves), whereas metamorph individuals lacked both gills and gill slits (Denoël 2017). Individuals undergoing metamorphosis were characterized by intermediate morphology, showing partial regression of external gills and progressive closure of gill slits compared with fully paedomorphic individuals. The exact stage of metamorphic progression could not be determined.

### Data acquisition and analyses

All following geometric morphometrics analyses, regression models, modularity, integration and disparity analyses, and their visualizations were performed in R statistical software (R Core Team v4.5.2).

#### Quantification of the body morphology and feeding apparatus shape

To quantify the body morphology, 16 linear measurements were taken from the external morphology using a digital caliper (Mitutoyo Absolute AOS Digimatic; Fig. 1B–D). The snout-vent length (SVL) was used as proxy for body size. All measurements were log10-transformed before performing analyses.

Micro-computed tomography (µCT) scanning was used to create 3D model of the bone’s morphology from the feeding apparatus. The scans were performed in three different imaging facilities. At the 3D Imaging Lab of the University of Tübingen, a Nikon XT H320 scanner with a tungsten reflection target was used with the following parameters: 190 kV, 100 µA, 19 Wms, 250 ms exposure time, and no filter. At the GISMO platform of the Université Bourgogne Europe, an RX Solutions Easytom S with the following parameters: 100 kV, 300 µA. with 0.5 mm aluminum filter. The scan used five images average in 12,5 im/s. At the Functional Anatomy and Vertebrate Evolution laboratory at the University of California, Berkeley using a GE Phoenix Nanotom M CT system with a 90 µA, 100 kV/20 W nanofocus tube. The voxel sizes used vary between 0.008 and 0.029 mm (Table S1).

The studied structures were segmented and extracted as PLY files using Avizo (v8.1, FEI Visualization Sciences Group). The cranium, mandible, and hyoid system were segmented separately. The resulting 3D models were imported into Geomagic Wrap (3D Systems, v2013.0.1) for mesh cleaning.

For a detailed and quantitative analysis of the feeding apparatus bones, we used 3D geometric morphometrics. Landmarks were digitalized using Checkpoint Stratovan (Stratovan Corporation, v23.04.07) on the left side of the cranium and mandible: (1) Cranium: 75 anatomical landmarks; (2) Mandible: 5 anatomical landmarks and 60 sliding semi-landmarks positioned on two curves (position of landmarks is detailed on Fig. 1E–G and Fig. S1; Tables S2 and S3). All landmarks were placed on homologous regions for each bone across life cycle categories. Bones undergoing resorption or formation during metamorphosis were excluded from the comparison between paedomorphs and metamorphs. Because ossification patterns in the hyoid system differ between life cycles strategies, homologous bones are not consistently ossified in paedomorphic and metamorphic individuals (Djorovic and Kalezic 2000; Heiss and Grell 2019; Schwarz et al. 2020). As a result, homologous landmarks cannot be placed, and we therefore compared life cycles by calculating the percentage of bone presence in each life cycle = (number of specimens with the bone/total number of specimens) × 100. All anatomical landmarks and semi-sliding landmarks were defined and manually collected by the same person (MF). The 3D coordinates of all landmarks were exported in IDAV Landmark Editor format in Checkpoint Stratovan software. To ensure spatial homology along curves, semi-landmarks were slid using the «slicer3d» function (Morpho package v2.13, Schlager 2017). Generalized Procrustes Superimposition (GPS) was performed on each 3D landmark dataset using the «procGPA» function (Morpho package v2.13, Schlager 2017) to obtain shape data. The centroid size of each bone structure (cranium and mandible) of each specimen was calculated using the « cSize » function (Morpho package v2.13, Schlager 2017). These centroid sizes were then log10-transformed and used as a proxy for size in allometric analyses.

#### Objective 1: Evaluating differences in allometric trajectories and morphological differentiation

##### Investigating the influence of size, life cycle, and locality on body and skull morphology

To explore the effect of size, life cycle, and their interaction, as well as the locality (defined by country), we performed multivariate analysis of covariance (MANCOVA) on the entire data, including metamorphs, paedomorphs, and specimens undergoing metamorphosis. First, models were fitted separately for each anatomical structure studied (body morphology, cranium, and mandible) using the « mvols » function (mvMORPH package v1.2.2; Clavel et al. 2015). Morphological traits were included as response variables, and life cycle categories (paedomorphs, metamorphs, paedomorphs undergoing metamorphosis) and size (SVL and centroid size) were included as predictors, as well as their interaction. Additionally, the locality factor was included as a covariate in all models. We used a penalized-likelihood approach (the “PL-LOOCV” method in “mvgls”- Penalized Log-Likelihood with Leave-One-Out Cross-Validation; Clavel et al. 2019) that can handle high-dimensional data (i.e., more variables than specimens). The significance of each predictor was assessed with partial tests (type II Pillai’s test statistic) and 999 permutations using the “manova.gls” function (mvMORPH package v1.2.2; Clavel et al. 2015). The predictors’ effect size for all the multivariate analyses was extracted using the “effectsize” function (mvMORPH package v1.2.2; Clavel et al. 2015).

We further investigated the morphological differences between life cycle categories using pairwise multivariate comparisons. These analyses were conducted on previous fitted models using the “pairwise.glh” function (mvMORPH package v1.2.2; Clavel et al. 2015). Statistical significance was also assessed using Pillai’s trace statistic with 999 permutations and Holm correction for multiple testing.

##### Testing for body size differences across life cycle

To assess the effect of life cycle category on body size, ordinary least squares (OLS) models were fitted using the function « lm » (stats package v4.5.2), with SVL as response variable and life cycle category as predictor and the locality factor as a covariate. A type II univariate analysis of variance (ANOVA) was performed on the fitted model using the function « Anova » (car package 3.1–3, Fox and Weisberg 2018). Pairwise comparisons among life-cycle categories were then performed using the “emmeans” function (emmeans package v2.0.1), with Holm correction.

##### Evaluating and visualizing differences in allometric trajectories depending on life cycle

To visualize allometric trajectories separately for each life cycle category, we fitted multivariate linear models similar to those described above for the MANCOVA analyses, but excluding the locality factor. Multivariate scores (mvscores) were then extracted from the fitted models using the “plot” function (mvMORPH package v1.2.2). These scores correspond to the projection of the multivariate shape data onto the regression vector associated with each predictor in the model (Drake and Klingenberg 2008). In this context, they represent the component of morphological variation explained by body size (SVL or centroid size), allowing visualization of life cycle-specific allometric trajectories. Predicted values from the multivariate regression models were projected onto the same plane to facilitate comparison of allometric trajectories among metamorphs, paedomorphs, and paedomorphs undergoing metamorphosis. The contribution of size to morphological variation within each life cycle category was assessed using specific contrast tests implemented in “manova.gls” (mvMORPH package v1.2.2). These specific analyses enabled us to test for allometry independently within each group. Additional contrasts were computed to test for differences in allometric slopes between life cycle categories, thereby evaluating whether developmental mode influences the direction of size-related shape variation (for access to the R code, please refer to the Data Availability Statement).

##### Visualizing shape variation across life cycle

To visualize the distribution of specimens according to life cycle type as well as their associated morphology in a morphological space, we performed a principal component analysis (PCA). We used the «prcomp» function for analyzing body morphology and «gm.prcomp» function (geomorph package 4.0.10, (Baken et al. 2021) for cranial and mandibular shape. Datasets included both metamorphic and paedomorphic individuals, as well as paedomorphic specimens undergoing metamorphosis. The contribution of each individual body morphological trait to the PCs (the loadings) was obtained using the « fviz_contrib » function (factoextra package 1.0.7, Kassambara 2017). For geometric morphometric analyses, we displayed the bone shape variation along each PC axis. To do this, a reference specimen, the individual closest to the center of the morphospace, was first selected (MNHN 1992–2685 for the cranium and MNHN 1992–2823 for the mandible). Then, the PCA projection corresponding to specimens with extreme scores (minimum and maximum) on each principal component (PC) axes were warped onto that reference shape using the « tps3d » function (Morpho package v2.13, Schlager 2017).

##### Investigating the influence of life cycle on the hyoid and coronoid bones

Because the remodeling of the hyoid bone during metamorphosis leads to differences in ossified elements between paedomorphic and metamorphic populations (Rose 2003), direct shape comparisons are not possible. Therefore, the presence of each ossified hyoid bone was quantified as a percentage within each life-cycle category (paedomorphic, metamorphic, and paedomorphic individuals undergoing metamorphosis). Similarly, the coronoid bone, which is resorbed during metamorphosis and disappears in fully metamorphosed newts, was scored as present or absent and expressed as a percentage across life-cycle categories.

#### Objective 2: Evaluate the effects of facultative life cycles on morphological integration, modularity, and disparity

##### Assessing morphological modularity and integration in relation to the life cycle

To test whether the different structures (body, cranium, and mandible) are more modular (i.e., whether parts of an anatomical structure covary more independently from other parts; Wagner 1996) in metamorphic populations than in paedomorphic populations, we conducted analyses of morphological integration and modularity separately for each morphological structure (body, cranium and mandible) in metamorphic and paedomorphic populations. Comparing integration and modularity between morphs therefore allows us to test whether alternative developmental trajectories are associated with different degrees of developmental constraint and independence among morphological traits. We used the functions « integration.test » and « modularity.test » (geomorph package v4.0.10; Adams and Otárola-Castillo 2013; Adams et al. 2022). These approaches quantify the covariance ratios within and among hypothesized modules and compare these ratios with null hypotheses of random assignments of shape variables to partitions (Adams and Felice 2014). Four modules were considered for the cranium, corresponding to early, mid and late development, and metamorphosis (Table S4). Two modules were defined for the mandible, separating the dentary, which undergoes partial remodeling, from the prearticular–angular complex. Two functional modules were identified for the body, representing locomotion-related and prey-capture regions. Modules considered for each structure are listed in Table S5. Both integration and modularity hypotheses were tested using 1000 permutations. The functions « compare.pls » and « compare.CR » were used to assess differences of modularity and integration values between life cycles.

##### Investigating morphological disparity in relation to the life cycle

Morphological disparity of the body, cranium and mandible was quantified using the sum of variances as a disparity metric for each paedomorphic and metamorphic group using the function « disparity.per.group » (dispRity package v1.3.5; Guillerme 2018). Disparity was evaluated for each entire structure (body, cranium and mandible) and also for different part of each structure. Pairwise differences in morphological disparity between life cycles were evaluated using Wilcoxon’s test of significance, employing the « test.dispRity » function (dispRity package v1.3.5; Guillerme 2018).

## Results

### Results for objective 1: Differences in allometric trajectories and morphology

#### Size, life cycle, and locality influence the morphology

The MANCOVA tests on the body morphology and the shape of each bone of the feeding apparatus (Table 1) show that life cycle, size (SVL or centroid size), locality, and the interaction between life cycle and size significantly affected all structures studied. Size is the factor most strongly associated with body morphology and cranial shape (effect size: body morphology = 0.908, cranium = 0.853). For mandibular shape, its effect is similar to that of life cycle (0.561 for life cycle and 0.578 for centroid size). Overall, life cycle shows a stronger association with morphology than locality. However, when estimated within the same model of cranial morphology, the effect sizes of life cycle and locality are similar (0.686 and 0.669, respectively).

**Table 1:** Results of the type II MANCOVAs tests on the relationship between body morphology or bone shape and size (SVL and centroid size), as well as life cycle categories.

Structure	Factor	Stat	*P-*value	Effect size
**Body morphology**	life cycle	0.936	**0.001**	0.423
	SVL	0.915	**0.001**	0.908
	Locality	0.582	**0.001**	0.231
	life cycle : SVL	0.356	**0.001**	0.108
**Cranium**	life cycle	1.697	**0.001**	0.686
	Csize	0.931	**0.001**	0.853
	Locality	1.687	**0.001**	0.669
	life cycle: Csize	1.310	**0.001**	0.262
**Mandible**	life cycle	1.515	**0.001**	0.561
	Csize	0.767	**0.001**	0.578
	Locality	1.336	**0.001**	0.381
	life cycle : Csize	1.222	**0.001**	0.277

*Note:* Significant *P-*values are indicated in bold.

Associated pairwise comparisons on the body morphology, cranial and mandibular shape show significant differences between metamorphic and paedomorphic populations (Table 2). Individuals undergoing metamorphosis differs significantly from paedomorphic individuals, but not from that of metamorphic ones.

**Table 2:** Results for pairwise MANCOVA tests for differences in body and feeding apparatus morphology between life cycle categories.

Structure	Test	Stat	*P*-value	Effect size
**Body morphology**	Metamorphic—paedomorphic	0.295	**0.003**	0.235
	Metamorphic—undergoing metamorphosis	0.046	0.909	0^a^
	Paedomorphic—undergoing metamorphosis	0.175	**0.012**	0.102
**Cranium**	Metamorphic—paedomorphic	0.718	**0.003**	0.396
	Metamorphic—undergoing metamorphosis	0.560	0.237	0.056
	Paedomorphic—undergoing metamorphosis	0.664	**0.003**	0.278
**Mandible**	Metamorphic—paedomorphic	0.677	**0.003**	0.397
	Metamorphic—undergoing metamorphosis	0.520	0.166	0.094
	Paedomorphic—undergoing metamorphosis	0.585	**0.042**	0.222

*Note*: Significant *P-*values are indicated in bold.

a Very small value that can be negative, indicating no association.

#### Life cycle and locality influence the body size

The results of the ANOVA indicate a significant effect of both life cycle and locality on size (Table 3). Associated pairwise comparisons show significant differences between metamorphic and paedomorphic populations, with metamorphic individuals being larger than paedomorphic ones (Table S5). Specimens undergoing metamorphosis show no difference in size (SVL) with paedomorphs, while the difference is significant with metamorphic individuals (Table 3).

**Table 3:** Results for type II ANOVA tests for differences in body size (SVL) according to life cycle categories, along with associated pairwise tests.

Type of test	Life cycle categories		Stat	*P*-value
**Global**	**All**	Life cycle	7.494	**<0.001**
		Locality	6.480	**0.002**
**Pairwise**	**Metamorphic—paedomorphic**		3.797	**<0.001**
	**Metamorphic—undergoing metamorphosis**		2.301	**0.045**
	**Paedomorphic—undergoing metamorphosis**		−0.311	0.756

*Note:* Significant *P-*values are indicated in bold.

#### Allometric trajectories differ among life cycle categories

Results of the contrast tests evaluating the contribution of size on morphology for each life cycle category show a significant effect for every structure (Table S6). The effect size associate to these contrast tests is higher in paedomorphic individuals and lower in individuals undergoing metamorphosis across all structures examined.

Tests comparing size–shape relationships among life cycle categories reveal significant differences between metamorphic and paedomorphic individuals for all morphological structures (Table S7). These differences in allometric trajectories are reflected in the slope estimates, which tend to be steeper in metamorphic individuals than in paedomorphs, indicating greater shape differentiation across the size range, particularly for cranial and mandibular structures (Fig. 2). Significant differences are also detected between paedomorphic individuals and those undergoing metamorphosis. No significant differences are found between metamorphic individuals and those undergoing metamorphosis for any of the structures studied.

**Fig. 2:**
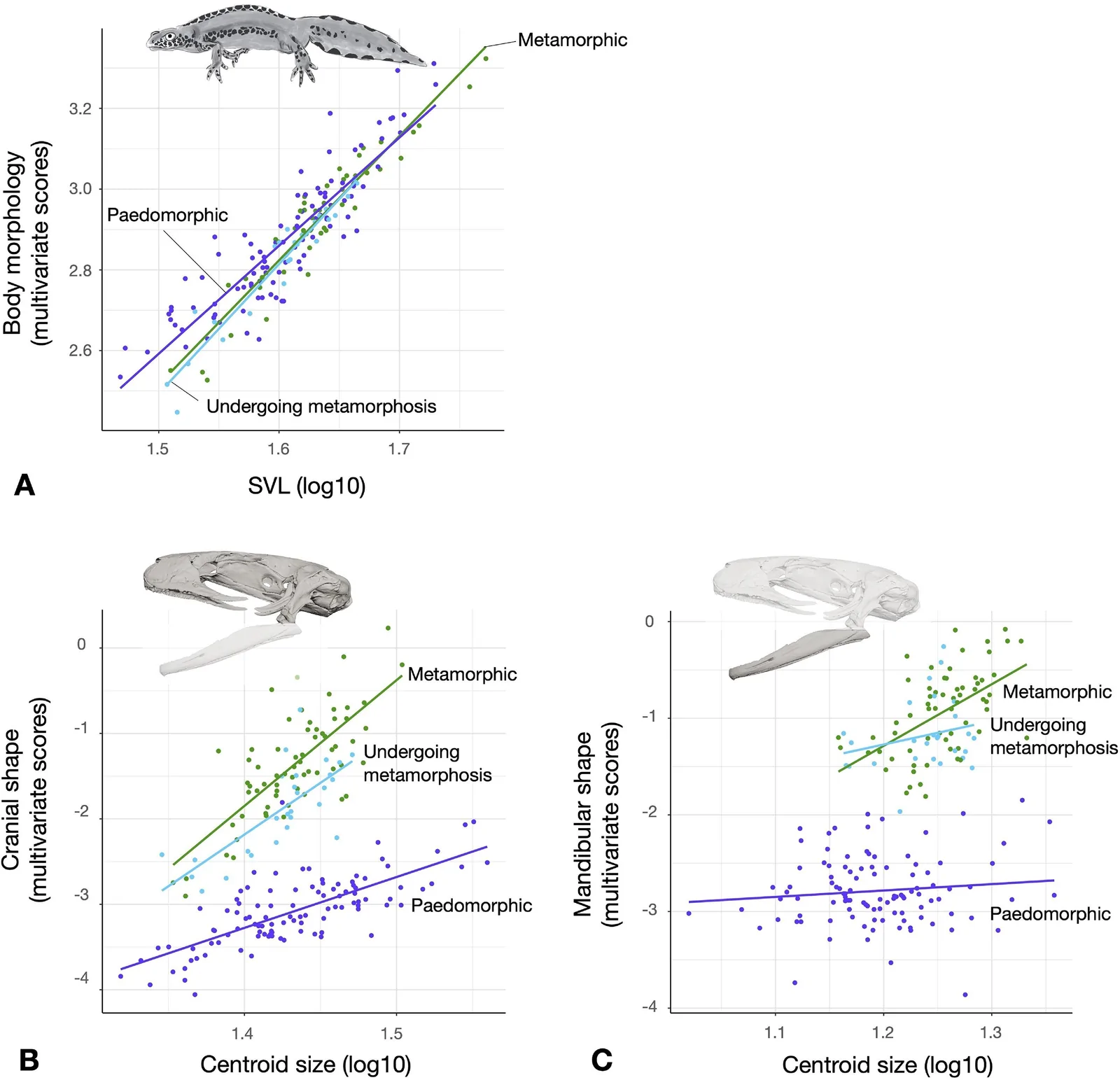
Allometric trajectories for body morphology (A), cranium (B) and mandible (C) across life-cycle categories.

#### Morphological differences throughout life cycle

The body morphology of paedomorphic and metamorphic individuals separate with some overlap along PC2 (13.3% of the overall variance, Fig. 3). The first axis mainly explains variation in body size (PC1 = 53.6%). Paedomorphic individuals tend to have larger heads in terms of length and height, as well as higher body height than metamorphic ones. Metamorphic individuals tend to have longer metacarpi and metatarsi than paedomorphic ones (Fig. 3).

**Fig. 3:**
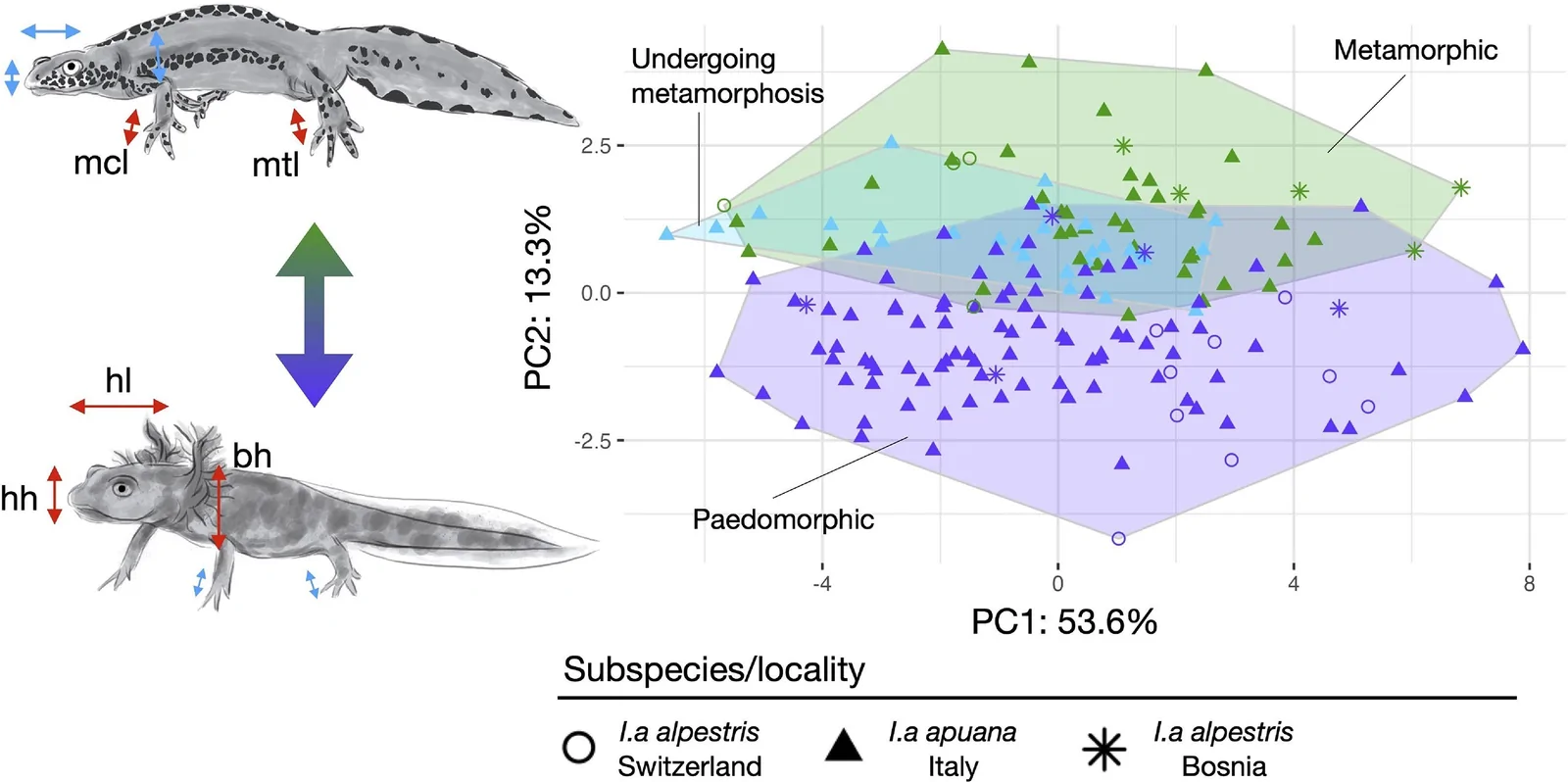
Morphospace of the body morphology obtained from the two first axes of the PCA. The differences across life cycle categories in the measurements that explain the most variance are illustrated in relation to the information stored in Fig. S2. Blue measurements are shorter and red measurements are longer. Abbreviations are as follow: hl: head length; hh: head height; bh: body height; mcl: metacarpus length; mtl: metatarsus length.

For the cranial shape, metamorphic individuals clearly separate from the paedomorphic ones on the first axis of the PCA (PC1 = 71.7% of the overall variance, Fig. 4A). In paedomorphic individuals, the maxilla is less developed, with a processus maxillaris less extended posteriorly. The vomer is less posteriorly developed. The postero-lateral processus of the frontal is less developed. The external naris is more laterally developed. The ventral processus of the squamosal and the distal articulation of the quadrate are more anteriorly and ventrally oriented. The distal articulation of the quadrate is less antero-posteriorly developed.

**Fig. 4:**
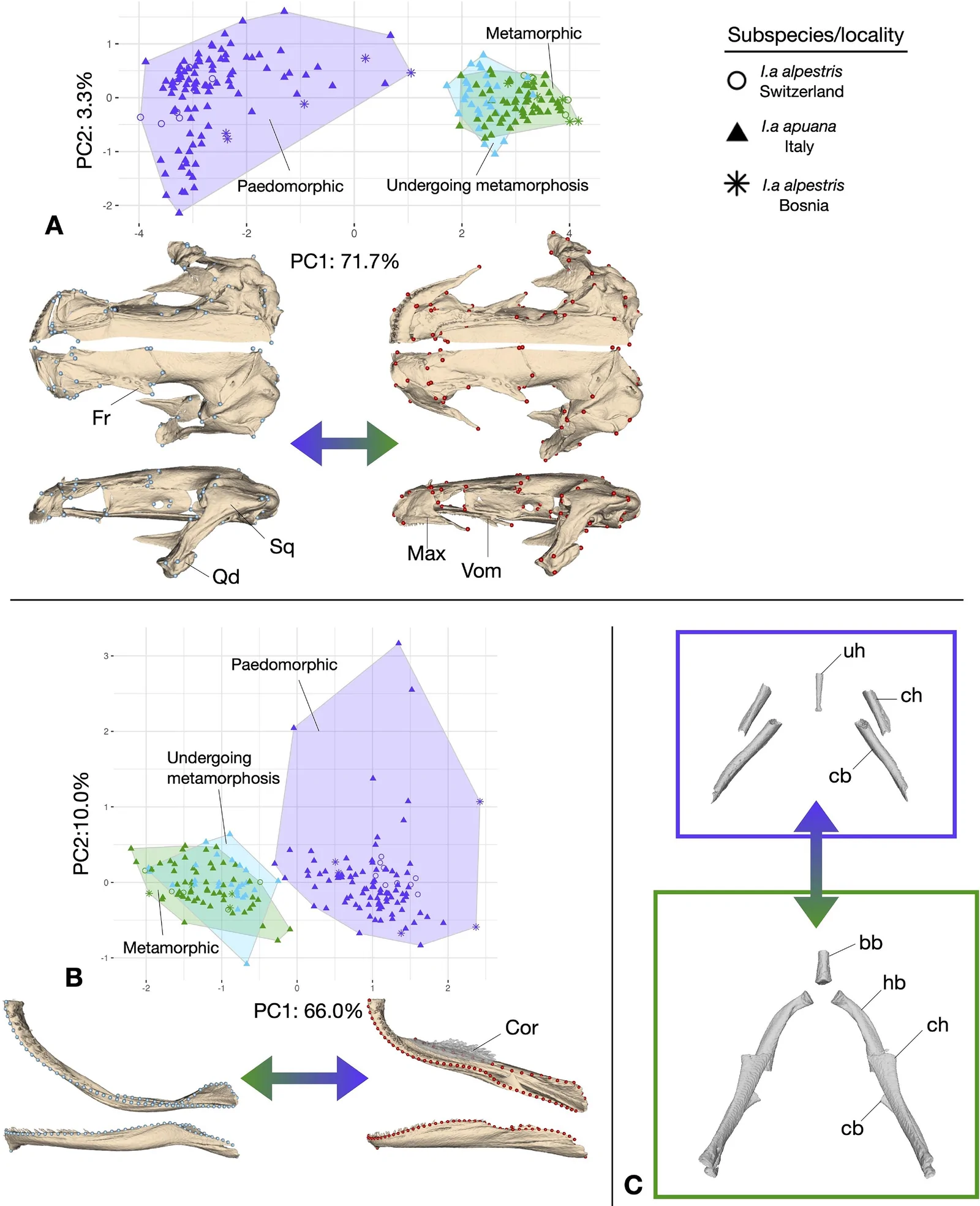
Morphospaces for the cranium (A) and mandible (B) obtained from the two first PCA axes as well as mesh illustration of the differences in ossified bones from the hyoid apparatus depending on life cycle (C). The 3D models illustrate morphological deformations applied to a reference specimen at the minimum (blue landmarks) and maximum (red landmarks) values along the first axis. In B, the bone in transparent gray indicates the coronoid, a bone that is not taken into consideration in the PCA. Abbreviations are as follow: bb: basibranchial; cb: ceratobranchial; ch: ceratohyal; hb: hypobranchial; Cor: coronoid; Fr: frontal; Max: maxilla; Qd: quadrate; Sq: squamosal; Vom: vomer.

For the mandibular shape, metamorphs separate from paedomorphs on the first axis (PC1 = 66.0%, Fig. 4B). Paedomorphic individuals have a straighter and more robust mandible in comparison to metamorphic ones. In addition, the coronoid (which is not included in the PCA and the other shape analyses) is present in 97% of the paedomorphic specimens, 2% of the specimens undergoing metamorphosis and in 4% of the metamorphic ones.

The percentage of hyoid bones presence is reported in Table 4 and ossified bones present in more than 70% of individuals are illustrated in Fig. 4C. In the hyoid apparatus of paedomorphic specimens, the urohyal is ossified in all individuals (100%). The ceratohyal and ceratobranchial I are ossified in 19% and 17% of specimens, respectively, whereas the basibranchial and hypobranchial I show very low levels of ossification (2% and 4%, respectively). In metamorphic specimens, the hypobranchial I is always ossified. The basibranchial, ceratohyal, and ceratobranchial I are ossified in 78%, 96%, and 89% of individuals, respectively, while the ceratobranchial II is ossified in only 4% of specimens. In paedomorphic specimens undergoing metamorphosis, the basibranchial, ceratohyal, hypobranchial I, and ceratobranchial I are ossified in 97%, 98%, 97%, and 98% of specimens, respectively. The hypobranchial II is rarely ossified, being present in only 3% of individuals. The urohyal is absent in both metamorphs and paedomorphs undergoing metamorphosis.

**Table 4:** Percentage of presence of ossified bones in hyoid apparatus depending on life cycle.

	Coronoid	Urohyal	Basibranchial	Ceratohyal	Hypobranchial 1	Hypobranchial 2	Ceratobranchial 1	Ceratobranchial 2
**Paedomorphic**	97%	100%	2%	19%	2%	0%	17%	4%
**Spe. undergoing metamorphosis**	2%	0%	97%	98%	97%	3%	98%	0%
**Metamorphic**	4%	0%	78%	96%	100%	0%	89%	4%

### Results for objective 2: Integration and modularity are conserved across life cycles, whereas disparity patterns differ

Pairwise comparisons reveal no significant differences in the level of modularity or integration between paedomorphic and metamorphic populations (Table 5).

**Table 5:** Values of integration (r.pls) and modularity (CR) depending on life cycle and comparison tests between paedomorphic and metamorphic populations.

		Integration			Modularity		
	Life cycle	r.pls	Effect size difference	*P*-value difference	CR	Effect size difference	*P*-value
**Body morphology**	**Metamorphic**	0.843	0.457	0.648	0.990	0.531	0.595
	**Paedomorphic**	0.783			0.930		
**Cranium**	**Metamorphic**	0.804	0.956	0.339	0.917	0.848	0.396
	**Paedomorphic**	0.874			0.917		
**Mandible**	**Metamorphic**	0.906	1.153	0.249	0.830	0.689	0.491
	**Paedomorphic**	0.948			0.848		

However, paedomorphic individuals tend to have higher disparity for the whole body, the entire cranium, and the entire mandible (Table S8; Fig. 5A). For the body parts, the legs and the head are more disparate in paedomorphic individuals while the trunk is more disparate in metamorphic ones (Fig. 5B, Table S8). Within the cranium, the frontal, maxilla, nasal, orbitosphenoid, otic-occipital, premaxilla, and vomer, exhibit a significantly higher disparity in paedomorphic populations. In contrast, the parietal, prefrontal, parasphenoid, pterygoid, quadrate and squamosal show significantly greater disparity in metamorphic populations (Fig. 5C; Table S8). Within the mandible, the dentary is significantly more disparate in paedomorphic individuals, while the prearticular-angular bone is more disparate in metamorphic ones.

**Fig. 5:**
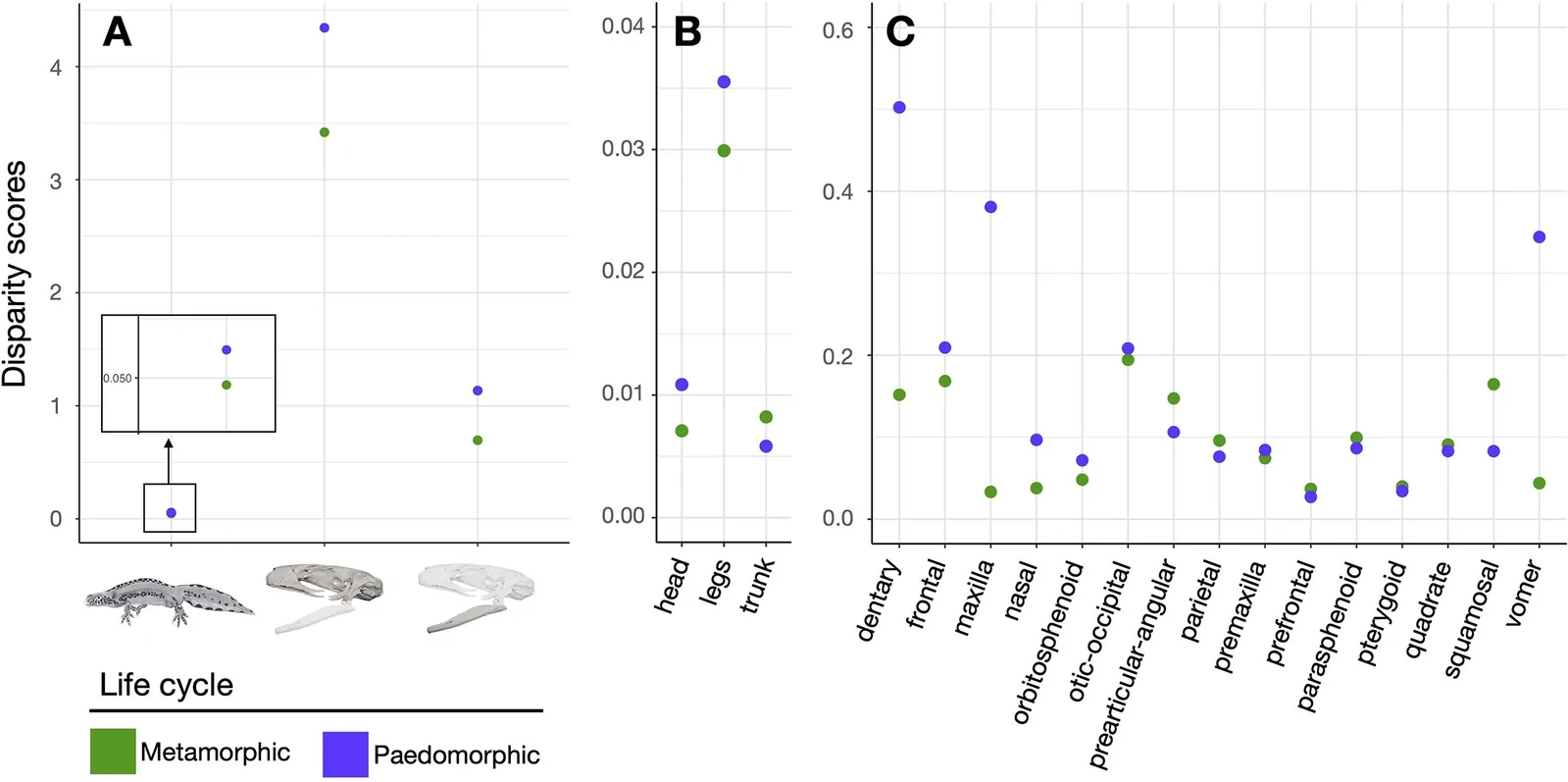
Disparity scores for entire structures (A), body regions (B), cranial and mandibular bones (C), obtained for each structure and life cycle.

## Discussion

Facultative paedomorphosis in the Alpine newt represents a remarkable example of developmental plasticity, allowing for the expression of facultative life cycle trajectories within and across populations (Denoël et al. 2005b; Whiteman 1994). Because metamorphic and paedomorphic individuals result from different heterochronic trajectories, they are expected to experience different developmental constraints (e.g., Bonett and Blair 2017). Paedomorphosis involves the retention of larval traits through these changes in developmental timing, whereas metamorphosis involves their modification or replacement during the transition to the terrestrial adult phenotype. These contrasting developmental pathways may consequently affect size, allometry, shape, the developmental independence of traits, and ultimately morphological disparity. Here, we investigate whether such developmental and associated functional (locomotion and feeding) constraints are reflected at the phenotypic level by examining how facultative paedomorphosis influences the size and morphology of the body and feeding apparatus in metamorphic, paedomorphic, as well as paedomorphic Alpine newts undergoing metamorphosis.

### Differences in size, morphology and allometric trajectories depending on life cycle type

As predicted, our results show that facultative paedomorphosis impacts the size, morphology and allometric trajectories of Alpine newts. More specifically, fully metamorphosed individuals are significantly larger than paedomorphic ones (Table 3; Table S5). This finding is consistent with previous observations showing that metamorphic individuals can be larger than paedomorphic individuals in some populations (Denoël et al. 2009). However, size differences between morphs are not consistent across populations: no significant difference in body size is also reported between paedomorphic and metamorphic individuals in others (Denoël et al. 2009). This suggests that the magnitude of morph-specific size differences may vary among populations. In our study, locality also had a significant effect on body size (Table 3), consistent with the idea that body size varies among populations and that local conditions may contribute to variation in size differences between morphs.

Size is corelated to the morphology of every studied structure, and allometric trajectories are associated with life cycle, showing that size affects morphology differently between metamorphic and paedomorphic populations (Fig. 2). Although metamorphic individuals exhibit a broader range of morphological variation between the smallest and largest specimens, the effect sizes in paedomorphic individuals are stronger (Table S6). This suggests that size accounts for a larger proportion of morphological variation in paedomorphs. In the Alpine newt, complete metamorphosis involves substantial restructuring of the feeding apparatus and associated functional shifts (Rose 2003; Ivanović et al. 2014; Schwarz et al. 2020). In contrast, aquatic paedomorphs undergo limited morpho-functional remodeling because they retain several larval features. The resulting conservation of larval morphology may constrain the extent to which cranial and mandibular shape can change with increasing body size, potentially explaining the stronger influence of size on morphological variation in paedomorphs.

Consistent with previous findings in Alpine newts (Ivanović et al. 2014; Heiss and Grell 2019), our analyses suggest that facultative paedomorphosis is associated with broad changes in the body morphology, as well as in cranial and mandibular shapes. Here, we show that paedomorphic individuals tend to have proportionally larger heads and smaller metapodial elements, whereas metamorphs exhibit longer metacarpi and metatarsi (Fig. 4). Paedomorphosis involves the retention of larval features, including external gills and head shape adaptations suited to an aquatic lifestyle, reflecting both the incomplete metamorphosis and the functional constraints imposed by the aquatic environment such as the suction-based feeding mode (Heiss and Grell 2019). A larger head in paedomorphs likely accommodates external gills and supports suction feeding mechanics by facilitating more powerful buccal expansion and proving larger areas for muscle attachment, which may enhance suction strike performance (Heiss and Grell 2019). At the same time, reduced metacarpi and metatarsi in paedomorphs likely reflect their reduced reliance on terrestrial locomotion. This is consistent with a broader pattern observed in salamanders, in which aquatic paedomorphs have distinct limb and digit proportions compared to terrestrial morphotypes (Ledbetter and Bonett 2019). Metamorphs, in turn, display relatively longer metacarpi and metatarsi, a pattern consistent with greater terrestrial mobility and the mechanical demands associated with substrate-based locomotion across heterogeneous terrestrial-aquatic habitats. It is important to note that the scatter and overlap observed in the morphospace in Fig. 3 likely reflect additional sources of variation, including population- or locality-level effects, as suggested by the significant effect of locality observed in our analyses (Table 1).

The larval traits in paedomorphs are also expressed in the morphology of the feeding apparatus, characterized by a less developed maxilla and vomer, the presence of coronoid bone in the lower jaw, and an ossified urohyal in the hyoid apparatus (Fig. 4). This pattern is consistent with the remodeling implied by metamorphosis (Rose 2003). Moreover, the quadrate is more developed ventrally. A more ventrally expanded quadrate may facilitate a larger mouth opening, enhancing suction performance. The more robust dentary observed in paedomorphs likely provides stronger attachment sites for muscles involved in the hyoid apparatus movements, further supporting efficient buccal expansion during suction feeding (Wake and Deban 2000). Moreover, paedomorphic Alpine individuals exhibit a less ossified hyoid apparatus, which likely increases the flexibility necessary for efficient suction feeding. The more cartilaginous structure allows rapid expansion of the buccal cavity, facilitating aquatic prey capture. In contrast, the more extensively ossified hyoid of metamorphic individuals provides the rigid support needed for tongue attachment, required for feeding on land (Deban and Wake 2000; Heiss and Grell 2019).

Individuals undergoing metamorphosis are similar in size to paedomorphs but remain significantly smaller than fully metamorphic newts (Table 3). Despite this similarity in size with paedomorphs, their body morphology and the shape of the feeding apparatus closely resemble those of metamorphic individuals (Figs 3 and 4; Table 2). These patterns are consistent with the central role of thyroid hormones in driving extensive tissue remodeling during metamorphosis (Rose 1996; Tata 1998, 2006) and suggest that morphological reorganization occurs before reaching an adult full body size in metamorphic individuals, resulting in a decoupling between size and shape evolution during metamorphosis. Rather than occupying an intermediate position, individuals undergoing metamorphosis cluster near the metamorphic morphology. This suggests that the shift in morphology relative to size occurs in a discrete manner once metamorphosis is initiated, rather than through a gradual, continuous allometric transition. Although the precise stage of metamorphosis could not be determined for each individual, and the degree of transformation may therefore vary among specimens, future studies including individuals at known developmental stages would be valuable to confirm whether morphological reorganization occurs early and in a discrete manner during metamorphic progression. Furthermore, the vast majority of individuals undergoing metamorphosis were sampled from a single locality. Because the evidence for a decoupling between morphological reorganization and metamorphic completion relies entirely on this group, additional populations should be examined to determine whether this pattern represents a general feature of facultative paedomorphosis or a response to local environmental conditions that triggered metamorphosis.

### Facultative paedomorphosis promotes disparate morphologies that are not related to a modification of developmental organization of traits

Our results suggest that variation in life cycle can decouple morphological divergence from changes in trait covariation at the intraspecific scale. Alternative developmental trajectories could theoretically modify developmental organization by altering the timing and coordination of growth among traits, thereby changing the developmental relationships among morphological traits during metamorphosis (Smith 2001; McNamara 2012; Klingenberg 2014). However, we found that the levels of morphological integration and modularity are similar between paedomorphic and metamorphic individuals (Table 5), indicating that alternative developmental trajectories do not substantially alter the developmental organization of the phenotype. Thus, the greater morphological disparity observed among paedomorphic individuals (Fig. 5A; Table S9) cannot be attributed to a greater independence among trait complexes or to a reorganization of their covariation structure. Instead, the similar patterns of integration and modularity suggest that paedomorphosis may generate morphological diversity without fundamentally altering the developmental relationships among traits. This partly contrasts with our second prediction and with previous interspecific studies showing that paedomorphic salamanders tend to exhibit more integrated feeding morphologies, whereas metamorphic species are more modular (Fabre et al. 2020; Louppe et al. 2025). The absence of differences at the intraspecific level suggests that shifts in developmental trajectory associated with facultative paedomorphosis may not be sufficient to substantially alter the underlying covariance structure among traits. One possible explanation is that paedomorphic and metamorphic morphs share the same genetic and developmental machinery, which may constrain variation in patterns of integration and modularity despite differences in morphology and allometric trajectories. Morphological integration and modularity arise from shared developmental, genetic, and functional interactions among traits, and these interactions are often conserved within species (Cheverud 1996; Young and Badyaev 2006; Klingenberg 2008; Singh et al. 2012). In contrast, pattern differences observed across salamander species (Fabre et al. 2020; Louppe et al. 2025) may have emerged over longer evolutionary timescales through the accumulation of developmental, genetic, and functional divergence. The contrasting patterns observed among salamander species (Fabre et al. 2020; Louppe et al. 2025) may therefore reflect deeper evolutionary differences in the processes shaping trait covariation, which are not necessarily recapitulated by the alternative developmental trajectories expressed within a species. Such differences may accumulate over longer evolutionary timescales as developmental, genetic, and functional relationships among traits diverge. Comparative evidence indeed shows that patterns of integration can be either conserved or labile across lineages, with changes in trait correlations accompanying morphological diversification in some clades (Watanabe et al. 2019; Mitteroecker and Bookstein 2008; Felice et al. 2019).

Our results show that paedomorphic populations exhibit greater morphological disparity than metamorphic populations across the body, cranium, and mandible (Fig. 5A; Table S9), consistent with previous findings at the interspecific level (Fabre et al. 2020; Louppe et al. 2025). Importantly, this greater disparity does not appear to be associated with differences in the developmental organization of traits, as paedomorphic and metamorphic individuals showed similar levels of morphological integration and modularity. The higher disparity observed in paedomorphic individuals may therefore arise from another source of variation. One possible explanation is inter-individual variation in the degree to which metamorphic development is expressed among paedomorphic individuals. Paedomorphosis does not necessarily terminate at the same developmental stage in all individuals, resulting in variation in the extent of metamorphic completion (Ivanović et al. 2014). Such variation should be particularly apparent in structures that normally undergo substantial remodeling during metamorphosis. Consistent with this hypothesis, differences in disparity between paedomorphic and metamorphic individuals were more pronounced for several cranial elements involved in metamorphic remodeling, including the vomer, maxilla, and dentary (Rose 2003; Fig. 5C; Table S8). During metamorphosis, the vomer undergoes substantial expansion and the maxilla develops and changes in morphology (), suggesting that variation in the degree of metamorphic development of these structures may contribute to the higher disparity observed among paedomorphic individuals.

Since partial metamorphosis promotes morphological diversity in the feeding apparatus, this could be reflected in the proportions of the head. However, the higher disparity observed in head proportions of paedomorphic Alpine newt may also reflect environmental effects. Gill surface area is known to vary with oxygen availability in the water (Duellman and Trueb 1994; Segev et al. 2019; Sabino-Pinto et al. 2019). Differences in oxygen concentration among Alpine newt populations could therefore promote increased morphological disparity.

Although morphological changes in the rest of the body are less pronounced during metamorphosis (Duellman and Trueb 1994), variation in the degree of metamorphosis can also affect limb morphology and contribute to a higher disparity. Further studies on paedomorphic newts integrating detailed environmental data are needed to disentangle developmental and environmental effects.

In contrast, some cranial and mandibular elements are more disparate in metamorphic individuals than in paedomorphs, including the prearticular-angular, parietal, quadrate, parasphenoid, and squamosal. These bones are associated with the opening and closing of the mouth through the insertion of the Musculus depressor mandibulae and the Musculus adductor mandibulae externus, internus, and posterior (Hinderstein 1971; Krogh and Tanner 1972; Carroll and Holmes 1980; Lorenz-Elwood and Cundall 1994; Deban and Wake 2000). This suggests that the diversity of feeding mode in metamorphs Alpine newts (Duellman and Trueb 1994; Thiesmeier and Schulte 2010; Heiss et al. 2013) may promote higher disparity in these elements compared with paedomorphs, which are largely constrained to suction feeding (Heiss and Grell 2019). However, this has only a minor impact on overall cranial morphology, which remains more disparate in paedomorphs.

## Conclusion

Despite the potential advantages of facultative paedomorphosis, the retention of larval traits in paedomorphic populations of the Alpine newt may impose developmental constraints that differ in strength and nature from those experienced by metamorphic individuals. Our analyses provide insight into how intraspecific variation in life cycle shapes morphological variation. Our results demonstrate that shifts in developmental timing associated with paedomorphosis influence morphology, allometric trajectories, and morphological disparity.

Paedomorphic individuals do not all interrupt metamorphic development at the same stage, resulting in variation in the extent of metamorphic completion. This variation may contribute to the higher morphological disparity observed in the body and feeding apparatus of paedomorphic individuals, particularly in structures that undergo substantial remodeling during metamorphosis. When metamorphic development resumes in facultative paedomorphs, individuals appear to rapidly approach the metamorphic size-shape trajectory, suggesting a relatively discrete rather than gradual morphological transition. Despite their greater morphological disparity, paedomorphic individuals retain a predominantly aquatic phenotype associated with aquatic feeding. This phenotype may enhance functional performance in stable aquatic habitats, but may also reduce ecological versatility compared with metamorphic individuals. Overall, our findings highlight how variation in developmental timing and the extent of metamorphic completion can shape morphology, allometric trajectories, and morphological disparity within species. Finally, paedomorphic populations are typically found in permanent, predator-free environments and are highly sensitive to disturbances such as the introduction of fish, habitat degradation, hydrological instability and climate change (Denoël et al. 2005a, 2016, 2019). Therefore, understanding how variation in life cycles impacts morphological patterns could provide valuable insights into their ecological vulnerability and potential resilience in the face of changing environmental conditions.

## Supplementary Material

icag164_Supplemental_File

## Data Availability

The data and R scripts used for the analyses presented in this study are available in the GitHub repository: *[Ichthyosaura_alpestris_life-cycle]* (https://github.com/M-Fournier/Ichthyosaura_alpestris_life-cycle).

## Notes

From the symposium “The evolutionary consequences of metamorphosis in metazoans” presented at the annual meeting of the Society for Integrative and Comparative Biology, January 3-7th , 2026.

## References

[bib1] Adams D, Collyer M, Kaliontzopoulou A, Baken E. 2022. Geomorph: Software for geometric morphometric analyses. R package version 4.0.4.

[bib2] Adams DC, Felice RN. 2014. Assessing trait covariation and morphological integration on phylogenies using evolutionary covariance matrices. PLoS One. 9:e94335. 10.1371/journal.pone.0094335. PMC3984176 24728003

[bib3] Adams DC, Otárola-Castillo E. 2013. Geomorph: an r package for the collection and analysis of geometric morphometric shape data. Methods Ecol Evol. 4:393–9. 10.1111/2041-210X.12035.

[bib4] Alberch P, Gould SJ, Oster GF, Wake DB. 1979. Size and shape in ontogeny and phylogeny. Paleobiology. 5:296–317. 10.1017/S0094837300006588.

[bib5] Bonett RM, Blair AL. 2017. Evidence for complex life cycle constraints on salamander body form diversification. Proc Natl Acad Sci USA. 114:9936–41. 10.1073/pnas.1703877114. PMC5604006 28851828

[bib6] Baken EK, Collyer ML, Kaliontzopoulou A, Adams Dean C. 2021. geomorph v4.0 and gmShiny: Enhanced analytics and a new graphical interface for a comprehensive morphometric experience. Methods in Ecology and Evolution. 12:2355–63. 10.1111/2041-210X.13723.

[bib7] Bonett RM, Ledbetter NM, Hess AJ, Herrboldt MA, Denoël M. 2022. Repeated ecological and life cycle transitions make salamanders an ideal model for evolution and development. Dev Dyn. 251:957–72. 10.1002/dvdy.373. 33991029

[bib8] Bonett RM, Steffen MA, Lambert SM, Wiens JJ, Chippindale PT. 2014. Evolution of paedomorphosis in plethodontid salamanders: ecological correlates and re-evolution of metamorphosis. Evolution. 68:466–82. 10.1111/evo.12274. 24102140

[bib9] Brown DD, Cai L. 2007. Amphibian metamorphosis. Dev Biol. 306:20–33. 10.1016/j.ydbio.2007.03.021. PMC1945045 17449026

[bib10] Carroll RL, Holmes R. 1980. The skull and jaw musculature as guides to the ancestry of salamanders. Zool J Linn Soc. 68:1–40. 10.1111/j.1096-3642.1980.tb01916.x.

[bib11] Cheverud JM . 1996. Developmental integration and the evolution of pleiotropy. American Zoologist. 36:44–50. 10.1093/icb/36.1.44.

[bib12] Clavel J, Aristide L, Morlon H. 2019. A penalized likelihood framework for high-dimensional phylogenetic comparative methods and an application to new-world monkeys brain evolution. Syst Biol. 68:93–116. 10.1093/sysbio/syy045. 29931145

[bib13] Clavel J, Escarguel G, Merceron G. 2015. mvMORPH: an R package for fitting multivariate evolutionary models to morphometric data. Methods Ecol Evol. 6:1311–9. 10.1111/2041-210X.12420.

[bib14] Deban SM, Marks SB. 2002. Metamorphosis and evolution of feeding behaviour in salamanders of the family Plethodontidae. Zool J Linn Soc. 134:375–400. 10.1046/j.1096-3642.2002.00004.x.

[bib15] Deban SM, Wake DB. 2000. Aquatic feeding in salamanders. In: Schwenk K editor. Feeding. Cambridge (MA): Academic Press. p.65–94. 10.1016/B978-012632590-4/50004-6.

[bib16] Denoël M . 2003. How do paedomorphic newts cope with lake drying?. Ecography. 26:405–10.

[bib17] Denoël M . 2017. On the identification of paedomorphic and overwintering larval newts based on cloacal shape: review and guidelines. Curr Zool. 63:165–73. 10.1093/cz/zow054 PMC5804162 29491974

[bib18] Denoël M . 2025. Insights from paedomorphic newts in introduced populations. NeoBiota. 99:249–63.

[bib19] Denoël M, Duguet R, Dzukic G, Kalezic M, Mazzotti S. 2001. Biogeography and ecology of paedomorphosis in *Triturus alpestris* (Amphibia, Caudata). J Biogeogr. 28:1271–80.

[bib20] Denoël M, Dzukic G, Kalezic ML. 2005a. Effects of widespread fish introductions on paedomorphic newts in Europe. Conserv Biol. 19:162–70.

[bib21] Denoël M, Ficetola GF. 2014. Heterochrony in a complex world: disentangling environmental processes of facultative paedomorphosis in an amphibian. J Anim Ecol. 83:606–15. 10.1111/1365-2656.12173 24180255

[bib22] Denoël M, Ficetola GF, Sillero N, Džukić G, L.Kalezić M, Vukov T, Muhovic I, Ikovic V, Lejeune B. 2019. Traditionally managed landscapes do not prevent amphibian decline and the extinction of paedomorphosis. Ecol Monogr. 89:e01347.

[bib23] Denoël M, Ivanović A, Džukić G, Kalezić ML. 2009. Sexual size dimorphism in the evolutionary context of facultative paedomorphosis: insights from European newts. BMC Evol Biol. 9:278. 10.1186/1471-2148-9-278 PMC2799415 19954520

[bib24] Denoël M, Joly P, Whiteman HH. 2005b. Evolutionary ecology of facultative paedomorphosis in newts and salamanders. Biol Rev. 80:663–71. 10.1017/S1464793105006858 16221333

[bib25] Denoël M, Joly P. 2000. Neoteny and progenesis as two heterochronic processes involved in paedomorphosis in *Triturus alpestris* (Amphibia: Caudata). Proc R Soc Lond B Biol Sci. 267:1481–5. 10.1098/rspb.2000.1168 PMC1690691 10983835

[bib26] Denoël M, Schmidt BR, Fonters R, Hansbauer G, Johanet A, Kühnis J, Poboljsaj K, Schweiger S, Sillero N. 2023. Quantifying rarity of intraspecific diversity at multiple spatial scales by combining fine-grain citizen-based data across national boundaries. Biol Conserv. 280:109937.

[bib27] Denoël M, Scime P, Zambelli N. 2016. Newt life after fish introduction: extirpation of paedomorphosis in a mountain fish lake and newt use of satellite pools. Curr Zool. 62:61–9. 10.1093/cz/zov003 PMC5804127 29491892

[bib28] Djorović A, Kalezić ML. 2000. Paedogenesis in European newts (Triturus: salamandridae): cranial morphology during ontogeny. J Morphol. 243:127–39. 10.1002/(SICI)1097-4687(200002)243:2<127::AID-JMOR2>3.0.CO;2-0. 10658197

[bib29] Doyle JM, Whiteman HH. 2008. Paedomorphosis in *Ambystoma talpoideum*: effects of initial body size variation and density. Oecologia. 156:87–94. 10.1007/s00442-008-0977-2. 18274781

[bib30] Drake AG, Klingenberg CP. 2008. The pace of morphological change: Historical transformation of skull shape in St Bernard dogs. Proc Biol Sci. 275:71–6. 10.1098/rspb.2007.1169 PMC2562403 17956847

[bib31] Drake AG, Klingenberg CP. 2010. Large‐scale diversification of skull shape in domestic dogs: Disparity and modularity. Am Nat. 175:289–301. 10.1086/650372. 20095825

[bib32] Duellman WE, Trueb L. 1994. Biology of Amphibians. Baltimore: JHU Press. 10.56021/9780801847806.

[bib33] Džukić G, Kalezić ML, Tvrtković N, Djorović A. 1990. An overview of the occurrence of paedomorphosis in Yugoslav newts (Triturus, Salamandridae) populations. Brit Herpetol SOC Bull. 34:16–22.

[bib34] Fabre AC, Bardua C, Bon M, Clavel J, Felice RN, Streicher JW, Bonnel J, Stanley EL, Blackburn DC, Goswami A. 2020. Metamorphosis shapes cranial diversity and rate of evolution in salamanders. Nat Ecol Evol. 4:1129–40. 10.1038/s41559-020-1225-3. 32572219

[bib35] Felice RN, Watanabe A, Cuff AR, Noirault E, Pol D, Witmer LM, Norell MA, O'Connor PM, Goswami A. 2019. Evolutionary integration and modularity in the archosaur cranium. Integr Comp Biol. 59:371–82. 10.1093/icb/icz052. 31120528

[bib36] Fox J, Weisberg S. 2018. An R Companion to Applied Regression. California: Sage Publications.

[bib37] Goswami A, Smaers JB, Soligo C, Polly PD. 2014. The macroevolutionary consequences of phenotypic integration: from development to deep time. Phil Trans R Soc B, 369:20130254. 10.1098/rstb.2013.0254. PMC4084539 25002699

[bib38] Guillerme T . 2018. DispRity: a modular R package for measuring disparity. Methods Ecol Evol. 9:1755–63. 10.1111/2041-210X.13022.

[bib39] Heiss E, Aerts P, Van Wassenbergh S. 2013. Masters of change: seasonal plasticity in the prey-capture behavior of the Alpine newt *Ichthyosaura alpestris* (Salamandridae). J Exp Biol. 216:4426–34. 10.1242/jeb.091991. 24259258

[bib40] Heiss E, Grell J. 2019. Same but different: aquatic prey capture in paedomorphic and metamorphic Alpine newts. Zoological Lett. 5:24. 10.1186/s40851-019-0140-4. PMC6660708 31372238

[bib41] Hinderstein B . 1971. The Desmognathine Jaw Mechanism (Amphibia: caudata: plethodontidae). Herpetologica. 27:467–76.

[bib42] Ivanović A, Cvijanović M, Denoël M, Slijepčević M, Kalezić ML. 2014. Facultative paedomorphosis and the pattern of intra- and interspecific variation in cranial skeleton: lessons from European newts (*Ichthyosaura alpestris* and *Lissotriton vulgaris*). Zoomorphology. 133:99–109. 10.1007/s00435-013-0202-7.

[bib43] Kassambara A . 2017. Factoextra: extract and visualize the results of multivariate data analyses. R package version 1.0.7.

[bib44] Klingenberg CP . 2008. Morphological integration and developmental modularity. Annu Rev Ecol Evol Sys. 39:115–32. 10.1146/annurev.ecolsys.37.091305.110054.

[bib45] Klingenberg CP . 2014. Studying morphological integration and modularity at multiple levels: concepts and analysis. Phil Trans R Soc B. 369:20130249. 10.1098/rstb.2013.0249. PMC4084535 25002695

[bib46] Krogh JE, Tanner WW. 1972. The hyobranchium and throat myology of the adult Ambystomidae of the United States and northern Mexico. Brigham Young Univ Sci Bull Biol Ser. 16:1–69.

[bib47] Larson A . 1980. Paedomorphosis in relation to rates of morphological and molecular evolution in the salamander Aneides flavipunctatus (Amphibia, Plethodontidae). Evolution. 34:1–17. 10.1111/j.1558-5646.1980.tb04785.x. 28563204

[bib48] Ledbetter NM, Bonett RM. 2019. Terrestriality constrains salamander limb diversification: implications for the evolution of pentadactyly. J Evol Biol. 32:642–52. 10.1111/jeb.13444. 30891861

[bib49] Lejeune B, Sturaro N, Lepoint G, Denoël M. 2018. Facultative paedomorphosis as a mechanism promoting intraspecific niche differentiation. Oikos. 127:427–39. 10.1111/oik.04714.

[bib50] Liedtke HC, Wiens JJ, Gomez-Mestre I. 2022. The evolution of reproductive modes and life cycles in amphibians. Nat Commun. 13:7039. 10.1038/s41467-022-34474-4. PMC9672123 36396632

[bib51] Lorenz Elwood JR, Cundall D. 1994. Morphology and behavior of the feeding apparatus in Cryptobranchus alleganiensis (Amphibia: caudata). J Morphol. 220:47–70. 10.1002/jmor.1052200106 29865388

[bib52] Louppe V, Le Verger K, Clavel J, Ferreira GS, Kyriakouli C, Stanley EL, Watt EC, Bardua C, Pérez-Ben C, Fröbisch NB et al. 2025. Complete metamorphosis promotes morphological and functional diversity in Caudata. Funct Ecol. 39:1732–47. 10.1111/1365-2435.70044.

[bib53] Lyons TA, Arbuckle K. 2024. The neoteny goldilocks zone: the evolution of neoteny in Ambystoma. Ecol Evol. 14:e11240. 10.1002/ece3.11240. PMC10999947 38590547

[bib54] McKinney ML, McNamara KJ. 1991. Heterochrony: The Evolution of Ontogeny. New York: Plenum Press. 10.1007/978-1-4757-0773-1.

[bib55] McNamara KJ . 2012. Heterochrony: the evolution of development. Evol Educ Outreach. 5:203–18.

[bib56] Mitteroecker P, Bookstein F. 2008. The evolutionary role of modularity and integration in the hominoid cranium. Evolution. 62:943–58. 10.1111/j.1558-5646.2008.00321.x. 18194472

[bib57] Moran NA . 1994. Adaptation and constraint in the complex life cycles of animals. Annu Rev Ecol Syst. 25:573–600. 10.1146/annurev.es.25.110194.003041.

[bib58] Okamiya H, Sugime R, Furusawa C, Inoue Y, Kishida O. 2021. Paedomorphosis in the Ezo salamander (Hynobius retardatus) rediscovered after almost 90 years. Zoological Lett. 7:14. 10.1186/s40851-021-00183-x. PMC8653548 34876208

[bib59] Patterson KK . 1978. Life history aspects of paedognic populations of the mole salamander Ambystoma talpoideum. Copeia. 1978:649–55. 10.2307/1443692.

[bib60] Renaud S, Alibert P, Auffray JC. 2012. Modularity as a source of new morphological variation in the mandible of hybrid mice. BMC Evol Biol. 12:141. 10.1186/1471-2148-12-141. PMC3506452 22873779

[bib61] Rolian C, Willmore KE. 2009. Morphological integration at 50: patterns and processes of integration in biological anthropology. Evol Biol. 36:1–4. 10.1007/s11692-009-9052-0.

[bib62] Rose CS . 1996. An endocrine–based model for developmental and morphogenetic diversification in metamorphic and paedomorphic urodeles. J Zool. 239:253–84. 10.1111/j.1469-7998.1996.tb05451.x.

[bib63] Rose CS . 2003. The developmental morphology of salamander skulls. In: Heatwole H, Davies M, editors. Amphibian Biology. Sydney: Surrey Beatty & Sons Pty Ltd. Vol. 5, p. 1684–781.

[bib64] Sabino-Pinto J, Goedbloed DJ, Sanchez E, Czypionka T, Nolte AW, Steinfartz S. 2019. The role of plasticity and adaptation in the incipient speciation of a fire salamander population. Genes. 10:875. 10.3390/genes10110875. PMC6896149 31683677

[bib65] Schlager S . 2017. Morpho and Rvcg–shape analysis in R: r-packages for geometric morphometrics, shape analysis and surface manipulations. In: Zheng G, Li S, Székely G, editors. Statistical Shape and Deformation Analysis. Cambridge (MA): Academic Press. p.217–56. 10.1016/B978-0-12-810493-4.00011-0.

[bib66] Schwarz D, Konow N, Porro LB, Heiss E. 2020. Ontogenetic plasticity in cranial morphology is associated with a change in the food processing behavior in Alpine newts. Front Zool. 17:34. 10.1186/s12983-020-00373-x. PMC7667761 33292303

[bib67] Segev O, Pezaro N, Rovelli V, Rybak O, Templeton AR, Blaustein L. 2019. Phenotypic plasticity and local adaptations to dissolved oxygen in larvae fire salamander (*Salamandra infraimmaculata*). Oecologia. 190:737–46. 10.1007/s00442-019-04446-5. 31250185

[bib68] Semlitsch RD, Harris RN, Wilbur HM. 1990. Paedomorphosis in *Ambystoma talpoideum*: maintenance of population variation and alternative life-history pathways. Evolution. 44:1604–13. 10.1111/j.1558-5646.1990.tb03849.x 28564302

[bib69] Singh N, Harvati K, Hublin JJ, Klingenberg CP. 2012. Morphological evolution through integration: a quantitative study of cranial integration in Homo, Pan, Gorilla and Pongo. J Hum Evol. 62:155–64. 10.1016/j.jhevol.2011.11.006. 22178399

[bib70] Smith KK . 2001. Heterochrony revisited: the evolution of developmental sequences. Biol J Linn Soc. 73:169–86. 10.1111/j.1095-8312.2001.tb01355.x.

[bib71] Tata JR . 1998. Amphibian metamorphosis as a model for studying the developmental actions of thyroid hormone. Ann Endocrinol. 59:433–42. 10189985

[bib72] Tata JR . 2006. Amphibian metamorphosis as a model for the developmental actions of thyroid hormone. Mol Cell Endocrinol. 246:10–20. 10.1016/j.mce.2005.11.024. 16413959

[bib73] Thiesmeier B, Schulte U. 2010. Der Bergmolch—im Flachland wie im Hochgebirge zu Hause. Bielefeld: Laurenti-Verlag.

[bib74] Vučić T, Sibinović M, Vukov TD, Tomašević Kolarov N, Cvijanović M, Ivanović A. 2019. Testing the evolutionary constraints of metamorphosis: the ontogeny of head shape in Triturus newts. Evolution. 73:1253–64. 10.1111/evo.13743 30990882

[bib75] Wagner GP . 1996. Homologues, natural kinds and the evolution of modularity. Am Zool. 36:36–43. 10.1093/icb/36.1.36.

[bib76] Wagner GP, Altenberg L. 1996. Complex adaptations and the evolution of evolvability. Evolution. 50:967–76. 10.1111/j.1558-5646.1996.tb02339.x. 28565291

[bib77] Wake DB, Deban SM. 2000. Terrestrial feeding in salamanders. In: Schwenk K., editior. Feeding: form, function and evolution in tetrapod vertebrates. Cambridge (MA): Academic Press. p.95–116. 10.1016/B978-012632590-4/50005-8.

[bib78] Watanabe A, Fabre AC, Felice RN, Maisano JA, Müller J, Herrel A, Goswami A. 2019. Ecomorphological diversification in squamates from conserved pattern of cranial integration. Proc Natl Acad Sci USA. 116:14688–97. 10.1073/pnas.1820967116. PMC6642379 31262818

[bib79] Whiteman HH . 1994. Evolution of facultative paedomorphosis in salamanders. Q Rev Biol. 69:205–21. 10.1086/418540.

[bib80] Wilbur HM . 1980. Complex life cycles. Annu Rev Ecol Syst. 11:67–93. 10.1146/annurev.es.11.110180.000435.

[bib81] Young RL, Badyaev AV. 2006. Evolutionary persistence of phenotypic integration: influence of developmental and functional relationships on complex trait evolution. Evolution. 60:1291–9. 10.1111/j.0014-3820.2006.tb01206.xa. 16892978

